# An Unusual Case of Acute Epiglottic Abscess

**Published:** 2014-01

**Authors:** Tanthry Deepalakshmi, Devan PP, Manjunath Prasad

**Affiliations:** 1 *Department of Otorhinolaryngology & Head & Neck Surgery, AJ Institute Of Medical Sciences, Mangalore– Udupi Highway, Kuntikana, Mangalore – 575004, Karnataka, India.*

**Fig 1:**
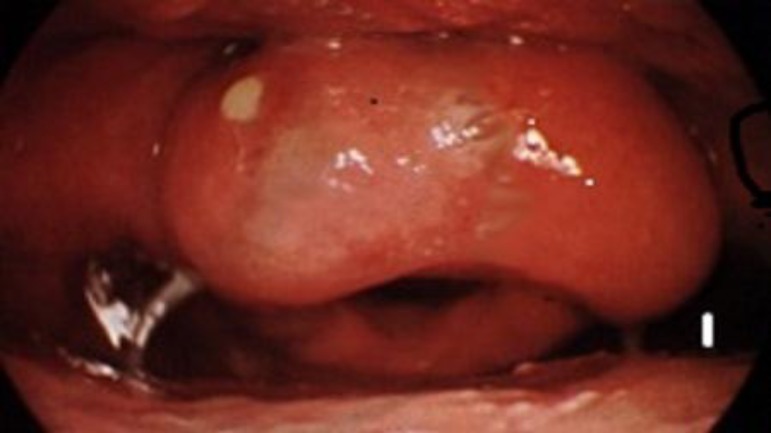
Pus Pointing Over Epiglottis

A 48 year old man presented to our outpatient department with the history of absolute dysphagia, and fever for two days. On examination patient was febrile with drooling of saliva. Oropharynx was minimally congested. Indirect laryngoscopy revealed odematous epiglottis with pus pointing over the lingual surface (Fig. 1).

**Fig 2:**
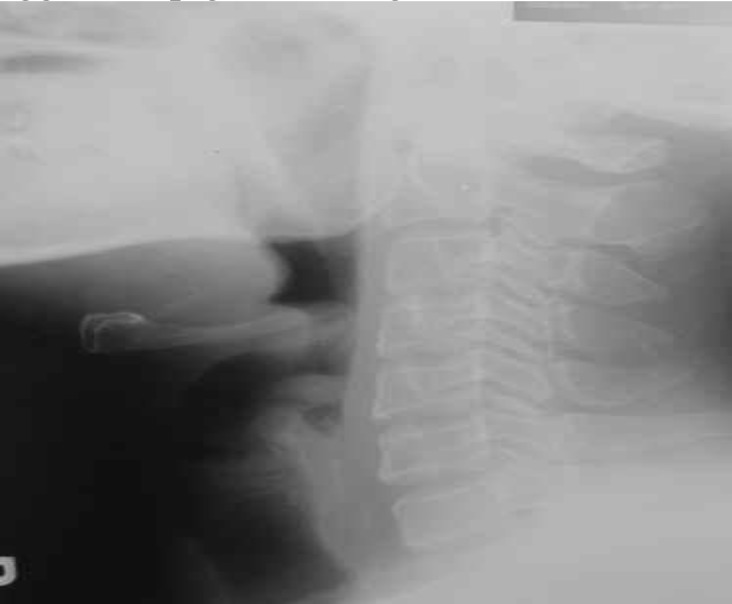
Radiography Shows the Thumb Sign

Radiography of soft tissue, neck lateral view, showed the thumb sign which suggested epiglottitis (Fig. 2). 

Incision and drainage was performed under general anaesthesia after haematological investigations. Patient was extubated the next day, and was discharged after two days, also oral antibiotics, and analgesics were prescribed. Patient was reviewed after 2 weeks, and indirect laryngoscopy revealed a normal epiglottis.

Although pharyngitis is the most common cause of sore throat in adults, acute epiglottitis must be considered in differential diagnosis when there is unrelenting throat pain, and minimal objective signs of pharyngitis. Epiglottic abscess formation is more common in adults than children. They most commonly occur as a complication of acute pharyngitis or with abscess of lingual tonsil .The abscess most frequently comes to a point on or near the lingual surface of the epiglottis. Streptococcus was isolated more frequently. Other organisms reported were Haemophilus influenzae, E.coli, Pseudomonas, Micro- coccus catarrhalis, Pneumococci. In our case, there were no preceding symptoms of acute pharyngitis. Risk factors include adult age at onset, diabetes mellitus, trauma, presence of a foreign body, and immune- compromised state. This case is unusual because of the absence of above risk factors. Incision and drainage under general anaesthesia is the treatment of choice. To the author^’^s knowledge, very few cases of acute epiglottic abscesses have been reported in the literature. This case is unusual because there are no preceding symptoms of pharyngitis or tonsillitis, and no association of risk factors like diabetes mellitus, trauma, foreign body or immunocompromised state.

